# The impact of teenage pregnancy on school dropout in Brazil: a Bayesian network approach

**DOI:** 10.1186/s12889-021-11878-3

**Published:** 2021-10-13

**Authors:** Emerson Cruz, Fabio Gagliardi Cozman, Wilson Souza, Albertina Takiuti

**Affiliations:** 1 Escola Politécnica, Universidade de São Paulo, São Paulo, Brazil; 2 Programa Saúde do Adolescente, Secretaria de Estado da Saúde de São Paulo, São Paulo, Brazil

**Keywords:** Teenage pregnancy, School dropout, Bayesian networks, Causality

## Abstract

**Background:**

As reported by the World Health Organization, adolescent pregnancy is a major public health concern given its impact on the life of mothers and their family members. In this study we investigated possible cause-effect relations between teenage pregnancy and school dropout, and other attributes that gravitate around them, using the Bayesian network approach.

**Methods:**

We used a database prepared by the Adolescent House Project and invited experts in the areas of Health, Education and Social Assistance to answer a survey containing questions aimed at detecting possible causal relationships. To perform the statistical analysis and the numerical simulations we employed the language and formalism of Bayesian networks.

**Results:**

The analysis indicated a strong cause-effect relation between teenage pregnancy and school dropout, bolstered by economic vulnerability. We were able to identify the profile of the female teenager who drops out from school: white girls older than 15 years who got pregnant at least once, are not working to generate an income, and who belong to the group where the family income is less than or equal to US$780 per month. Also we detected the “maternal impact factor", i.e., the effect caused by whether or not the mothers of the teenagers have experienced teenage pregnancy.

**Conclusion:**

There are many factors that lead teenagers to drop out of school; we confirmed not only the commonsense notion that pregnancy of the teenager is a major factor but found that a history of teenage pregnancy on the part of the mother is a major factor. Moreover, Bayesian networks emerged as an interesting mathematical framework to perform the statistical analysis.

## Background

### The big picture

It is well known that teenage pregnancy has varied negative impacts on teenager life, not only on health, but also, among others, on self esteem and on social and educational experience. Even though such consequences of teenage pregnancy are global concerns, some countries have felt them more intensely [1–5]. For example, Latin American countries face worrying numbers of teenage pregnancy; in Brazil, about five hundred thousand teenage pregnancies were estimated in 2019 by the Brazilian Society of Pediatrics [6]. Thus one should expect several government programs to be dedicated to this theme in Brazil.

One of these programs is the Adolescent Health Program of the State of São Paulo. This program supports the services provided by the *Casa do Adolescente* (Adolescent House) facilities, where a teenager receives help from multiple professionals in cross-functional teams (physicians, nurses, psychologists, and teachers, among others). The Adolescent Houses have operated for decades in a daily routine dedicated to the integral health of teenagers [7].

It is a commonsense observation that one of the many possible impacts of unplanned teenage pregnancy is school dropout [8–10]. However, such a complex phenomenon requires a detailed statistical analysis as there are several social/economic/health interacting variables whose spurious correlations and false cause-effect relations must be avoided. Moreover, for obvious reasons, unplanned pregnancies do not allow for randomized controlled experiments, which is why many studies have pursued observational methods. This can lead to a lack of distinction between correlations and possible causal relationships.

The main challenge we face is to build a mathematical model that combines numerical data with expert opinions so as to run a causal analysis around attributes associated with teenage pregnancy and school dropout. To do so, we resort to the Bayesian network approach, a methodology developed in the field of Artificial Intelligence with applications ranging from medicine [11] to decision-making support systems [12]. In short, we obtained the dependence and independence relations expressed by a Bayesian network from expert opinions, and the probabilities in the Bayesian network from data collected by the *Casa do Adolescente*. This approach should be useful in further exploring the factors that affect school dropout dynamics.

In the following sections we first summarize the necessary background on Bayesian networks and on recent tools that aim at causal inference with Bayesian networks; we then describe the data collection and the modeling procedures. After that, we present our results and offer some discussion and conclusions.

### Structural models and Bayesian networks

The first step in exploring possible causal relationships within a system of interest is to build a so-called Structural Causal Model (SCM). Basically, a SCM is a conceptual model that describes the relationships between the variables present in the system. More formally, the SCM can be understood as a set of functions related to two sets of variables: exogenous (U) and endogenous (V). Exogenous variables are external to the model, which means we choose not to explain then, while endogenous variables must be dependent on at least one exogenous variable. For example, the SCM in Expression (1) may describe a model where *X* and *Z* are exogenous variables while *Y* and *W* are endogenous variables related to *X* and *Z* by a set of equations *F*:

1 $$  \begin{aligned} U &= \{X,Z\}, \\ V &= \{Y,W\},\\ F &= \{W=f(Y,Z), Y=g(X)\}. \end{aligned}  $$

The Bayesian network (BN) formalism offers a visual representation for SCMs, one that has been applied to a variety of fields. In medicine, for example, Bayesian networks have been applied to problems related to medical diagnoses [11, 13]. A Bayesian network represents a probability distribution over a set of variables [14–16], where a variable might mean “is pregnant,” “has low income,” or “has dropped out of school.”

**Fig. 1:**
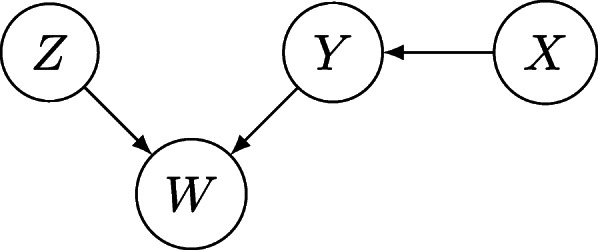
An example of a directed acyclic graph, where *W*, *X*, *Y* and *Z* are random variables; *X* and *Z* are exogenous variables while *Y* and *W* are endogenous variables

A Bayesian network consists of two components: a Direct Acyclic Graph (DAG) and a set of conditional probability tables associated with each node present in the graph. The graph is the qualitative part that represents the conditional probabilistic dependencies (arcs) between the variables (nodes), while the Conditional Probability Table (CPT) indicate the numerical values of those probabilistic dependencies. The DAG and the CPTs must satisfy a so-called Markov condition: a random variable must be independent of its non-descendants non-parents given its parents in the graph. Recall that the parents of a node *X* are those nodes such that arcs (or edges) emanate from them and end at *X*; the descendants of *X* are those nodes that are reached from *X* by following directed arcs, and the non-descendants of *X* are simply those nodes that are not descendants of *X*. An acyclic directed graph is depicted in Fig. 1.

This scheme induces a joint distribution for the *n* random variables in the graph as conveyed by Expression (2):

2 $$ {}P(X_{1}=x_{1}, \ldots X_{n}=x_{n}) = \prod_{k=1}^{n} P \left(X_{k}=x_{k} \mid \text{pa}(X_{k})=\pi_{k}\right),   $$

where pa(*X*_*k*_) denotes the set of parents of *X*_*k*_ and *π*_*k*_ denotes the projection of $\{x_{1},\dots,x_{n}\}$ onto pa(*X*_*k*_). If a variable *X*_*k*_ has no parents, then we take its corresponding term to be the unconditional probability *P*(*X*_*k*_=*x*_*k*_).

For instance, given the network in Fig. 1, we have:

3 $$ {}\begin{aligned} P(W&=w,X=x,Y=y,Z=z)\\ &= \mathrm{P}(X=x)\mathrm{P}(Z=z) \mathrm{P}(Y=y|X=x)\\ &\quad\ \mathrm{P}(W=w|Y=y,Z=z). \end{aligned}  $$

### The do-operator and the average causal effect

**Fig. 2:**
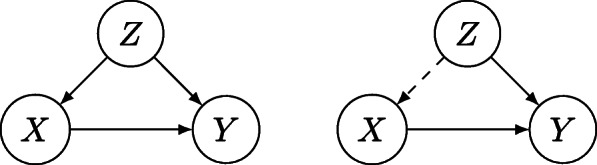
Intervention on X and adjustment for Z; X,Y and Z are random variables

To investigate whether a particular circumstance leads to another, say whether teenage pregnancy leads to school dropout, one must go beyond statistical correlations. Cause-effect questions can be addressed by carrying out interventions in assumed structural equations [17]. For example, consider the setup illustrated by Fig. 2. The so-called *do*-operator allows us to consider the effect on *Y* of intervening in *X* by “cutting” the edge from *Z* to *X*, thus obtaining:

4 $$ {}P(Y=y|do(X=x)) = {\sum_{z} P (Y = y | X = x, Z = z)P(Z=z) }.   $$

Expression (4) is referred to as an *adjustment formula*; it captures the connection between the variable *X* and *Y* for each particular value of *Z* and then averages over those values [16]. These calculations can be done using estimates from observational data; hence the do-operator offers a mathematical way of calculating the effect of an intervention from a graph and data-based estimates.

A metric that is associated with such operations is the Average Causal Effect (ACE):

5 $$ {}ACE = P(Y=y_{i}|do(X=x_{i}))- P(Y=y_{i}|do(X=x_{j})).   $$

As an example, consider the graph in Fig. 2. Denoting by *X* the binary variable related to *teenage pregnancy*, by *Y* the binary variable related do *dropping out the school* and by *Z* the binary variable related to *age*, one can analyze say the extent to which dropping out of school is impacted by being pregnant or not. To do so, the relevant ACE is *P*(*Y*=0|*do*(*X*=1))−*P*(*Y*=0|*do*(*X*=0)).

Bayesian networks offer a powerful formalism that can express subjectivity in phenomena where causality plays a central role [18, 19], while providing concrete mathematical models to perform statistical inference [20]. Also they are explainable and transparent, desiderata that we must satisfy in our setting.

## Methods

As noted in the previous section, a Bayesian network consists of two parts: its graph, often referred to as its “structure”, and its associated probabilities. The former captures the dependence and independence relations in the domain; the latter carries assessments and beliefs about such dependences. We start by describing how we collected data so as to estimate probability values, then we describe how we collected expert opinions so as to build the underlying graph. The analysis presented later, to be done using the do-operator and the ACE [17], depends both on the probability values and the graph.

### Data management

We used a database prepared by the *Projeto Casa do Adolescente* (Adolescent House Project) and available at 10.5281/zenodo.2633222. Data were collected in nineteen units distributed in eighteen cities in the State of São Paulo, Brazil, resulting in 343 teenagers (294 girls and 49 boys) interviewed with twenty nine questions. We selected eight of those questions that were aligned with the focus of our work. We take the adolescence as the period from 12 to 18 years of age in accordance to the criteria adopted by the Brazilian Child and Adolescent Statute [21].

### Attributes

The attributes (variables or nodes in the Bayesian network) selected for this work are:

- *Age*: The age of the person in years.
- *Ethnic Group (EG)*: This attribute is the respondent’s own declaration about which ethnicity he/she identifies with. We distinguish white or non-white.
- *Teenage Pregnancy (TP)*: This attribute is the number of pregnancies, including abortions, experienced by the respondent.
- *Maternal Impact Factor (MF)*: This attribute indicates whether the respondent’s mother has experienced teenage pregnancy.
- *School Enrollment Status (SS)*: This attribute indicates whether or not the respondent was enrolled in school at the time the survey was run.
- *Economic Status (ES)*: This attribute captures the monthly income of the respondent’s family, as declared by the respondent. At the time of this study (2019), the Brazilian minimal wage per month was, approximately, US$260.
- *Labor Status (LS)*: This attribute indicates whether or not the respondent had a job at the time the survey was done.

We also invited experts from Health, Education and Social Assistance to answer a survey containing questions aimed at mapping the perception of these experts about the possible causal relationships between the selected attributes. From the invited group, thirteen of them responded to the invitation (three Medical Doctors, three Nurses, four Psychologists, two Teachers and one Pedagogue). The questions of the survey are available at http://doi.org/10.5281/zenodo.3358177 and the resulting data set is available at http://doi.org/10.5281/zenodo.3358169.

The use of information and the collection protocols for these databases was authorized by the official institutions involved and approved by the Ethics Committee of São Paulo University Medical School (CEP-FMUSP) in full compliance with Resolutions No.466 (12/12/2012) and No.510 (07/04/2016) of the National Health Council (CONEP).

### Data processing

In order to perform the analysis we processed the data as follows:

- Missing Data Protocol: for all attributes, in a case of missing data we used the corresponding median as substitute.
- Data Exclusion Policy: considering the small percentage of male respondents (14.3%) compared to female respondents, we decided to focus the analysis only on the female group. Another reason for this decision is that teenage pregnancies have generally more impact on girls than on the boys. By doing so the total number of respondents dropped to 294.
- Balancing: With respect to the attribute “number of pregnancies experienced by the respondent (including abortions)”, we randomly selected the girls from the complete group so as to build two groups with the same number of participants (one group with girls who had experienced at least one pregnancy or abortion and another one who had not). Two groups were produced with 130 girls each.
- Binarization: All attribute values were converted to binary form to better suit the subsequent Bayesian analysis.
  - For the attribute “Age” we chose 15 years of age (the median of the period) as the dividing line.
  - For the “Ethnic Group” (EG) attribute, “White” and “Non-white” groups were produced (in accordance with the self declaration given by the respondents themselves).
  - For the “Economic Status” (ES) attribute, the dividing value was US$780, which corresponds to, approximately, three times the value of the Brazilian Minimum Wage at the time the study was run (2019). This particular value was the median of the data informed for this attribute.
  - For the “Pregnancy in Adolescence” (TP) attribute, we created a group with girls who had at least one pregnancy (including abortion) and another group with those who did not.
  - For the “Maternal Impact Factor” (MF) attribute, we produced a group where the mother of the respondent experienced at least one teenage pregnancy (including abortions) and another group where the mother did not experience teenage pregnancy.
  - For the attribute “School Enrollment Status” (SS), the respondents were separated into a group with the girls with regular school enrollment and another group with girls that dropped out the school.
  - Finally, for the attribute “Labor Status” we divided the respondents into a group where the teenagers were in the job market and another group where they were not.

The resulting list of binary attributes and their corresponding abbreviations are shown in Table 1.

**Table 1:** Attributes and their encoded binary values

Attribute	Abbreviation	0	1
1 - Age	Age	<15 years	≥15 years
2 - Ethnic Group	EG	White	Non-White
3 - Teenage Pregnancy	TP	No	Yes
4 - Mother Impact Factor	MF	No	Yes
5 - Economic Status	ES	< US$780/month	≥ US$780/month
6 - School Enrollment Status	SS	No	Yes
7 - Labor Status	LS	No	Yes

## Results

### Statistical description

A summarized statistical description of attributes in the dataset is depicted in Table 2. One can note that most teenagers are older than 15 years, suffering from economic vulnerability and without a regular job (actually this profile matches the general profile observed in the Adolescent House Project).

**Table 2:** Descriptive statistics

Attribute	Percentage distribution	
Age	0	30%
	1	70%
Ethnic Group (EG)	0	48%
	1	52%
Teenage Pregnancy (TP)	0	50%
	1	50%
Maternal Impact Factor(MF)	0	43%
	1	57%
Economic Status (ES)	0	76%
	1	24%
School Enrollment Status (SS)	0	34%
	1	66%
Labor Status (LS)	0	84%
	1	16%

The 50/50-distribution of teenage pregnancy is the result of balancing the data set

### Eliciting the structure of the Bayesian network

**Fig. 3:**
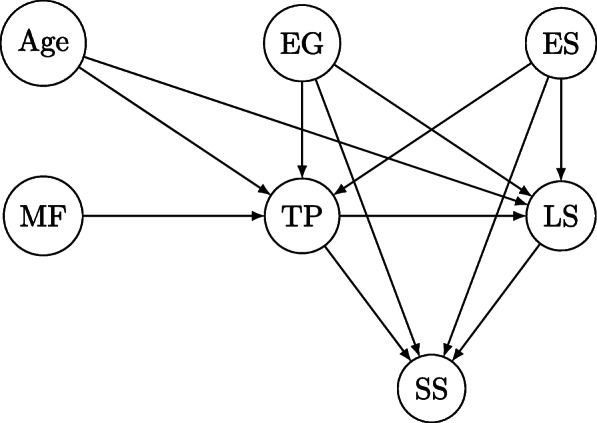
The structure of the Bayesian network. Abbreviations are explained in Table 1

To complete the Bayesian network used in this study, we combined the data described previously with expert opinions about dependences amongst variables. Experts were asked, through the survey we mentioned before, to indicate when an edge could be drawn from a particular variable to another one, interpreting this as a causal connection from the first to the second variable; the consensus result is depicted in Fig. 3.

The Pearson’s correlation factors, shown in Table 3, were directly computed from observed correlations in our dataset. It is important to pay attention to their signs as we are interested in understanding the connections amongst variables. For instance, the correlation between TP and SS is negative, meaning that teenage pregnancy makes it less likely to be in school.

**Table 3:** Correlations Factors

	Age	EG	TP	MF	ES	SS	LS
**Age**	**1**	SC	0.29	SC	SC	-0.17	0.25
**EG**		**1**	-0.01	SC	-0.15	0.02	0.01
**TP**			**1**	0.07	-0.22	-0.5	-0.04
**MF**				**1**	SC	SC	SC
**ES**					**1**	0.18	0.17
**SS**						**1**	-0.08
**LS**							**1**

SC stands for spurious correlation and abbreviations are explained in Table 1

Simulations and calculations with the Bayesian network were run using the software GeNie Modeler (https://www.bayesfusion.com/genie/).

### Estimating the average causal effects

Computations were run using the Bayesian network shown in Fig. 3. The results are described in Table 4. Calculations were done using Expressions (4) and (5). For instance, consider the ACE produced by the difference between states 1 and 0 of the teenage pregnancy attribute (TP):

6 $$ {}ACE = P(SS=0|do(TP=1)) - P(SS=0|do(TP=0)).   $$

**Table 4:** ACE values of interventions

Structure	Graph	Adjusted for	ACE
(a)		Age	0.44
(b)		ES	0.47
(c)		EG	0.48

Abbreviations are explained in Table 1

This expression assumes a surgical cut on the graph, simulating an intervention in teenage pregnancy; that is, it assumes a constant state for pregnancy and evaluates the effect on school dropout.

We employed Expression (4) to compute adjustments grouping the data according to the following criteria: age, economic status (ES) and ethnic group (EG). Table 4 shows the most relevant results. Adjusting for age we get *ACE*=0.44 which clearly indicates that being pregnant does not favor being enrolled in school. One can infer that teenage pregnancy leads to school dropout. By grouping the total population according to Economic Status (ES), we obtain *ACE*=0.47, again giving a strong indication that the occurrence of teenage pregnancy leads to school dropout. Finally, adjusting with respect to Ethnic Group (EG) and calculating the *ACE* we obtain 0.48, again suggesting that teenage pregnancy leads to school dropout. Hence, results indicate teenage pregnancy as a clear cause for school dropout in all scenarios in Table 4.

With regard to the effect of family history in teenage pregnancy (captured by the Maternal Impact Factor), calculations yielded a 58% probability of teenage pregnacy given teenage pregnancy of the mother, a high probability for the propagation of teenage pregnancy from mother to daughter. This result indicates that teenage pregnancy is a phenomenon that continues from generation to generation (in accordance with the informal opinions usually voiced by experts), suggesting that preventive actions and campaigns should be aimed not only at non-pregnant adolescents, but should also help mothers better plan their interactions with daughters.

## Discussion

Overall, the analysis of causal effects using the structure and probabilities in our Bayesian network agrees with several studies carried out in different locations around the world and with different methodologies [1, 10, 22–24]. Several of them have found a deleterious effect of teenage pregnancy on school dropout; we saw that in Brazil the same conclusion is warranted.

However, there are other factors that can also lead to school dropouts, such as early presence in the labor market motivated by the need to collaborate with family income. With our model it is reasonable to ask, as we did, what is the effect of family history on teenage pregnancy. As another example of query that might be posed, one might ask, among the groups (Age, ES, EG), which one is the group with the highest probability of dropping out of school due to teenage pregnancy?

Answers to such questions are not easy to obtain using simple statistics, particularly the last one where a large number of variables are involved. The Bayesian network approach offers useful machinery to address such questions. As an example, suppose we wish to find a profile for the “typical” teenager that drops out of school We can look for the most likely profile of the teenager who drops out of school, considering all the attributes we have. Assuming as evidence SS = No, the Maximum a Posteriori Probability (MAP) is attained by the following scenario: Age= ≥15yrs, EG=White, ES= <US$780, LS=No, MF=Yes, TP=Yes. That is, we obtain probabilistic evidence rooted in data and expert opinions to the extent that this typical subject is a white girl over the age of 15 years with a family income below US$780 per month, who is not currently working to generate income, and where both the mother and the teenager herself have experienced a teenage pregnancy.

Unplanned pregnancy negatively affects both maternal physical and psychological health, even increasing the demand for abortions in clandestine clinics and the consequently increasing the mortality rate of mothers due to unsafe abortions, especially in developing countries [25, 26]. In addition, teenage pregnancy is usually associated with lack of information and access to programs and services dedicated to teenager sexual and reproductive health, which further amplifies socioeconomic vulnerabilities and even loss of social identity [27–30]. Thus, public health and education policies aimed at both adolescent mothers and their children are essential not only to provide support and to mitigate negative consequences after the occurrence of an unplanned pregnancy, but also to act in reducing the socioeconomic vulnerabilities and social isolation in which adolescents may live [31]. Public decision making regarding prevention policies related to drop out must then take into account socioeconomic vulnerability, family history related to teenage pregnancy, labor status.

Furthermore, a Bayesian network lets one simulate interventions, for instance examining counterfactual questions — something we did not pursue in this paper [17]. Counterfactual reasoning would address questions such as: What would happen to the school enrollment status of a girl who is already pregnant if she did not have this experience of teenage pregnancy?

## Conclusion

Teenage pregnancy and school dropout are, unfortunately, problems faced by a huge portion of the world population. Hence it is not a surprise that these problems have received intense scrutiny in the scientific literature. Themes related to teenage pregnancy are usually analyzed under the perspective of health, while school dropout is discussed within education. However, these topics are strongly interconnected, particularly in scenarios where inequalities and social vulnerabilities are present.

In this study, we connected health and education by building a model capturing both dependence and independence relations, and associated probabilities. The resulting Bayesian network lets us consider hypothetical scenarios, a task that is essential for the design of public policies. Among several other insights, our results indicate a strong cause-effect relation between teenage pregnancy and school dropout, bolstered by economic vulnerability, in the settings where we collected our data. We also found that the most likely profile of a teenager dropping out of school is a white girl over the age of 15 years with a family income below US$780 per month, who is not currently working to generate income, and where both the mother and the teenager herself have experienced a teenage pregnancy. Results indicate that girls whose mothers experienced pregnancy at teenage age are more likely to repeat this phenomenon, thus suggesting that teenage pregnancy is a phenomenon that continues from generation to generation.

This study, of course, has its limitations; for example, related to the small data setting and the specificities of the environment in which the data were collected. However, the aim of this work was not to obtain a precise numerical description but to present an alternative approach to perform statistical analysis and investigate possible cause-effect relations in the context of teenage pregnancy. As far as we know, the machinery of Bayesian-network-based causal analysis has not been applied to the discussion of school dropout. Our efforts should be helpful as a road map to inspire further investigations, in particular extending the model with more attributes, and debating whether the structure of the Bayesian network we found from experts really represents the phenomena of interest.

## Data Availability

All the data sets used in this work can be found at [10.5281/zenodo.2633222], [http://doi.org/10.5281/zenodo.3358177] and [http://doi.org/10.5281/zenodo.3358169] all referenced in the main body of the text as well.

## Abbreviations

The abbreviations used in this work are listed below and can also be found throughout the main body of the text: .
ACE: Average causal effect
BN: Bayesian network
DAG: Direct acyclic graph
CPT: Conditional probability table
EG: Ethnic group
ES: Economic status
LS: Labor status
MAP Maximum a posteriori probability; MF: Maternal impact factor
SCM: Structural causal model
SS: School enrollment status
TP: Teenage pregnancy
